# *CK2*beta gene silencing increases cell susceptibility to influenza A virus infection resulting in accelerated virus entry and higher viral protein content

**DOI:** 10.1186/1750-2187-3-13

**Published:** 2008-07-23

**Authors:** Henju Marjuki, Christoph Scholtissek, Hui-Ling Yen, Robert G Webster

**Affiliations:** 1 Division of Virology, Department of Infectious Diseases, St. Jude Children's Research Hospital, Memphis, TN 38105, USA; 2 Department of Pathology, University of Tennessee, Memphis, TN 38105, USA

## Abstract

**Background:**

Influenza A virus (IVA) exploits diverse cellular gene products to support its replication in the host. The significance of the regulatory (β) subunit of casein kinase 2 (CK2β) in various cellular mechanisms is well established, but less is known about its potential role in IVA replication. We studied the role of CK2β in IVA-infected A549 human epithelial lung cells.

**Results:**

Activation of CK2β was observed in A549 cells during virus binding and internalization but appeared to be constrained as replication began. We used small interfering RNAs (siRNAs) targeting CK2β mRNA to silence CK2β protein expression in A549 cells without affecting expression of the CK2α subunit. *CK2*β gene silencing led to increased virus titers, consistent with the inhibition of CK2β during IVA replication. Notably, virus titers increased significantly when CK2β siRNA-transfected cells were inoculated at a lower multiplicity of infection. Virus titers also increased in cells treated with a specific CK2 inhibitor but decreased in cells treated with a CK2β stimulator. CK2β absence did not impair nuclear export of viral ribonucleoprotein complexes (6 h and 8 h after inoculation) or viral polymerase activity (analyzed in a minigenome system). The enhancement of virus titers by CK2β siRNA reflects increased cell susceptibility to influenza virus infection resulting in accelerated virus entry and higher viral protein content.

**Conclusion:**

This study demonstrates the role of cellular CK2β protein in the viral biology. Our results are the first to demonstrate a functional link between siRNA-mediated inhibition of the CK2β protein and regulation of influenza A virus replication in infected cells. Overall, the data suggest that expression and activation of CK2β inhibits influenza virus replication by regulating the virus entry process and viral protein synthesis.

## Background

Protein kinase CK2 is a pleiotropic, proliferation-associated, highly conserved and constitutively active Ser/Thr protein kinase found in the nucleus and cytoplasm of most cells [1-5]. CK2 is a tetramer comprising 2 functional catalytic subunits (α,α', or both) and 2 regulatory β subunits, which combine to form an αα'β_2_, α_2_β_2_, or α'_2_β_2 _heterotetramer [2,6-8]. CK2 is involved in many signal transduction pathways and in negative regulation of apoptosis [8-10]. It appears to play crucial roles in cell cycle and growth control via phosphorylation of several important substrates, including p53 [11], Cdc2 [12], and BRCA1 [13]. Mice lacking CK2α have lethal cardiac neural tube structural defects, highlighting the specific role of CK2α in the development of these organs [14]. Male CK2α'-knockout mice have defective spermatogenesis and are sterile [15]. CK2β is essential for cell viability in mice and is therefore required during early embryonic development [6].

The β-subunit of CK2 is trifunctional: (1) it stabilizes the heterotetrameric holoenzyme [16,17]; (2) it increases enzyme activity [18-20]; and (3) it determines substrate specificity [17,21]. Structure-function studies *in vitro *support a direct link between CK2 activity and the phosphorylation status of the β-subunit [22,23]. Using mass spectrometry analysis, Arrigoni et al. recently identified 144 different cellular proteins that interact with CK2β, 10% of which are involved in signaling pathways [24].

Type A influenza virus (IVA), belonging to the family *Orthomyxoviridae*, is highly contagious and causes devastating outbreaks in humans and several animal species. Its genome comprises 8 single-strand, negative-sense RNA molecules that encode as many as 11 viral proteins. These RNA segments are associated with nucleoproteins (NPs) and 3 RNA-dependent RNA polymerase subunits (PB1, PB2, and PA) to form viral ribonucleoprotein (RNP) complexes, which are the minimal infectious viral structures. The viral genome is replicated and transcribed in the host cell nucleus, requiring bidirectional transport through the nuclear membrane via nuclear pore complexes [25]. Because of the limited coding capacity of their genome, IVAs extensively employ host cellular mechanisms, including many different signaling pathways, to promote their replication [26-29].

IVAs appear to have acquired the capacity to either repress or exploit cellular responses that are upregulated during viral infection. For example, expression of the β-isoform of the protein kinase C superfamily (PKCβII) and activation of the Raf/MEK/ERK signal cascade, appears to promote IVA replication. PKCβII is a specific regulator of influenza virus entry into late endosomes [30,31], whereas phosphorylation of ERK is required for efficient nuclear export of viral RNP complexes [29,32]. Further, inhibition of PKC by the protein kinase inhibitor H7 alters the phosphorylation pattern of the NP protein [33] and causes retention of the late viral mRNAs in the nucleus [34]. In addition, activation of the nuclear factor NF-κB in response to IVA infection is partially suppressed by the viral NS1 protein, presumably to prevent excessive expression of interferon β (IFNβ), while at the same time the virus appears to use the remaining NF-κB activity for apoptosis-related and virus-supporting processes [26,35].

Sanz-Ezquerro et al. [36] demonstrated that the PA polymerase protein of IVA is phosphorylated at serine and threonine residues in cultured cells. Moreover, PA protein is a substrate of CK2α *in vitro*, suggesting that this kinase is involved in covalent posttranslational modification of PA. Other viruses have been shown to interact with this cellular kinase as well. CK2 phosphorylation of the P protein (subunit of the RNA-dependent RNA polymerase complex) of vesicular stomatitis virus is essential for its activity in viral transcription [37,38], and CK2 phosphorylation sites are found in the P protein of measles virus [39].

Although the significance of CK2β in various cellular mechanisms is well established, less is known about its role in IVA replication. We studied the involvement of CK2β at different stages of viral infection in human epithelial lung A549 cells. We used small interfering RNAs (siRNAs) specifically targeting CK2β mRNA to diminish CK2β protein expression and studied the effect on virus titers, virus internalization, viral protein synthesis, nuclear export of viral RNP complexes, and viral polymerase activity.

## Results

### CK2β is not activated during influenza virus replication

**Figure 1:**
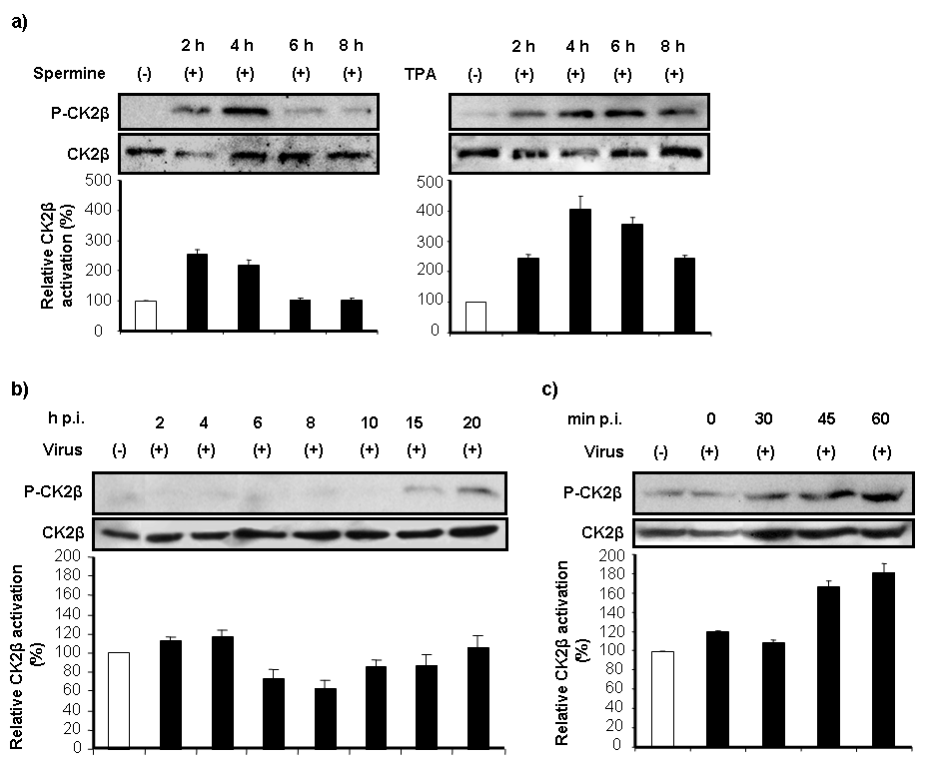
**Timing of CK2β activation**. (a) A549 cells were treated with either spermine (300 μM) or TPA (200 ng/ml) and further incubated for the times as indicated. (b) A549 cells were incubated with virus-containing medium (MOI = 5) at 37°C for 1 h, medium was replaced, and cells were further incubated at 37°C for the times as indicated. (c) A549 cells were incubated with virus-containing medium (MOI = 5) at 4°C for 20 min and then at 37°C for the times as indicated. CK2β activation was assessed at the indicated time points by Western blot with polyclonal rabbit anti-phosphorylated CK2β (P-CK2β). Bands in three independent experiments were quantified, and relative CK2β activation (black bars) was calculated and normalized to the loading control (anti-CK2β mAb). White bars represent uninoculated cells.

To demonstrate that CK2β can be activated in A549 cells, we assessed CK2β activation at different time points after stimulation with spermine (a polyamine that regulates CK2 activity through direct interaction with the β subunit of the holoenzyme [40]) or with the broad kinase activator 12-O-tetradecanoyl-phorbol-13-acetate (TPA). In spermine-stimulated cells, CK2β activation was increased at 2 h and 4 h but had returned to the basal level at 6 h and 8 h (Figure 1a, left). TPA treatment caused stronger kinase activation than did spermine, as compared to the respective controls. TPA-induced CK2β activation reached its highest level at 4 h and had progressively decreased at 6 h and 8 h (Figure 1a, right). These results show that CK2β can indeed be activated in the cell line used for this study.

To assess whether CK2β is activated by influenza virus, we incubated A549 cells with medium containing the human A/Puerto Rico/8/34 influenza virus (multiplicity of infection [MOI] = 5) for 1 h at 37°C and then analyzed CK2β activation at different time points by using an antibody that detects serine phosphorylation at position 209 of CK2β. CK2β activation in uninoculated cells was assigned a value of 100% (baseline control). CK2β activation did not differ between inoculated and control cells at any point during hours 2 to 20 after inoculation (Figure 1b), suggesting that CK2β is not activated during influenza virus replication.

We next assessed CK2β activation during the 1-h inoculation period, when virus binds to cells and is internalized. Cells were incubated with virus-containing medium (MOI = 5) at 4°C for 20 min. The medium was then replaced with pre-warmed medium and cells were incubated at 37°C. CK2β activation was analyzed 0, 30, 45, and 60 min after start of inoculation period. At 45 min and 60 min after infection, CK2β activation was approximately 65% and 80%, respectively, greater than that in control cells (Figure 1c). Therefore, CK2β activation is upregulated very early in the viral life cycle and is inhibited when virus replication starts.

### Inhibition of CK2β expression increases influenza virus titers

To evaluate the effect of CK2β on IVA replication, we silenced CK2β expression in A549 cells by using a gene-specific siRNA. To ensure that the amount of siRNA duplex used did not negatively influence cell viability, we first performed a cell proliferation assay 24 h, 48 h, and 72 h post-transfection (p.t.). CK2β siRNA did not reduce the number of viable cells or affect cell morphology, as compared to those of control cells, at any time point (data not shown), suggesting that the amount of siRNA used is not toxic to the cells.

**Figure 2:**
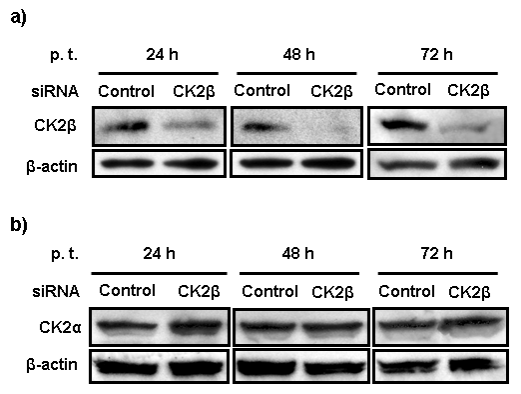
**Expression of CK2β protein**. A549 cells were transfected with either control or CK2β siRNA. (a) Total amount of CK2β protein detected at 24 h, 48 h, and 72 h post-transfection (p.t.) by Western blot analysis with a mAb specific for the β-subunit of CK2. Loading was controlled with a mAb against β-actin. (b) Total CK2α protein detected 24 h, 48 h, and 72 h after transfection with CK2β siRNA.

**Figure 3:**
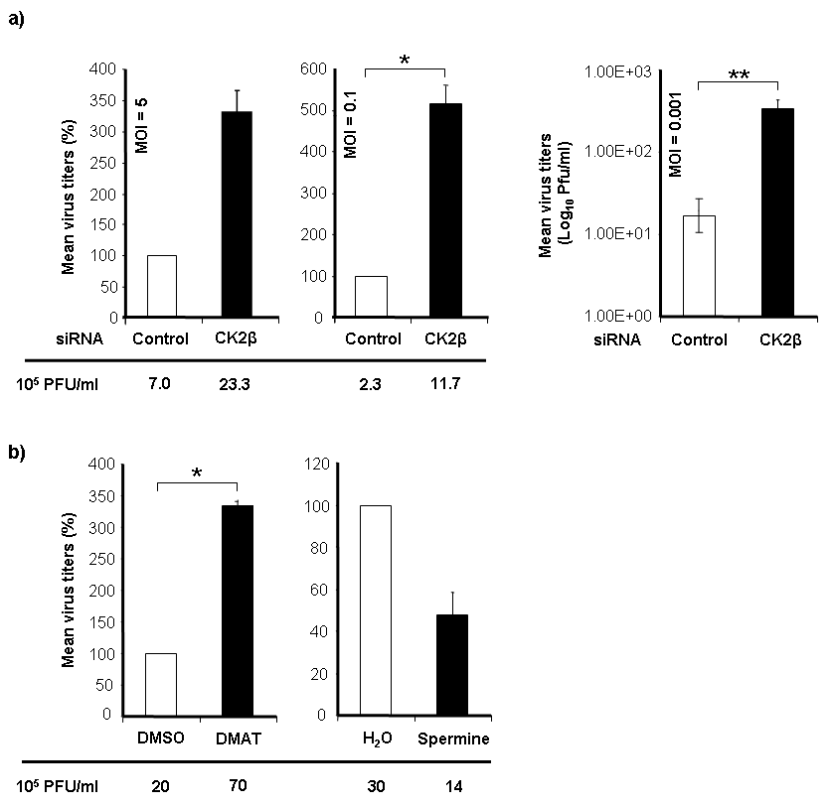
**siRNA-mediated silencing of CK2β increases virus titers**. (a) A549 cells were transfected with either control (white bars) or CK2β (black bars) siRNA for 48 h, then infected at an MOI of 5, 0.1, or 0.001 and incubated for 24 h. Virus in cell supernatants was titrated in MDCK cells. (b) A549 cells were inoculated with virus (MOI = 5) and were either left untreated (white bars) or treated with the CK2 inhibitor DMAT or the activator spermine (black bars). Virus in cell supernatants was titrated at 24 h p.i.. Mean virus titers ± SE from 3 independent experiments are given as a percentage of the control mean and as PFU/ml. * *P *< 0.05 and **P < 0.005 between the indicated groups.

Total amount of the CK2β protein was assayed 24 h, 48 h, and 72 h p.t. by Western blot analysis. In cells transfected with CK2β siRNA, CK2β expression was substantially less than that in control-transfected cells at 24 h p.t. and was almost completely absent at 48 h p.t. (Figure 2a), demonstrating the efficiency of this gene-specific siRNA. The amount of CK2β protein started to increase again at 72 h p.t. (Figure 2a), indicating that siRNA-mediated inhibition of CK2β gene expression is transient. Additionally, to determine whether CK2β gene silencing negatively affects expression of the α-subunit of CK2, we assayed CK2α protein levels at the same 3 time points. CK2α expression was not reduced in CK2β siRNA-transfected cells at 24 h, 48 h, or 72 h p.t. (Figure 2b). Because CK2β expression was most significantly reduced 48 h p.t., cells were inoculated with virus 48 h p.t. and further incubated for 24 h. At first, we used a relatively high MOI of 5, as A549 cells are less susceptible to IVA infection than commonly used cell lines such as MDCK cells. It is noteworthy that most of the cells remained viable after a 24-h inoculation at this MOI. Virus titers in the cell supernatants differed substantially 24 h post-inoculation (p.i.). The mean virus titer in CK2β siRNA-transfected cells was 3 times that in control siRNA-transfected cells (Figure 3a). The difference in virus titers was more pronounced when cells were inoculated with a very low virus dose (MOI = 0.1 and 0.001). At MOI = 0.1, the mean virus titer was approximately 5 times as high in cells transfected with CK2β siRNA as in control siRNA-transfected cells (Figure 3a). At MOI = 0.001, the difference exceeded 1 log10 (Figure 3a).

We next confirmed that CK2β activity influences the outcome of viral infection. To assess the effect of CK2 inhibition, we incubated cells with 5 μM 2-dimethylamino-4,5,6,7-tetrabromo-1H-benzimidazole (DMAT, a potent and specific CK2 inhibitor [41]) or with solvent (DMSO) before and during virus inoculation (MOI = 5). To determine the effect of CK2β activation, we treated cells with 300 μM spermine or with H_2_O before and during virus inoculation. At 24 h p.i., the mean virus titer in DMAT-treated cells increased as compared to controls (Figure 3b). Conversely, cells treated with the CK2β activator spermine had a mean virus titer approximately half that of the control titer at 24 h p.i. (Figure 3b). These results again suggest that activation of CK2β is inconsistent with efficient virus replication.

### Inhibition of CK2β expression accelerates influenza virus internalization

**Figure 4:**
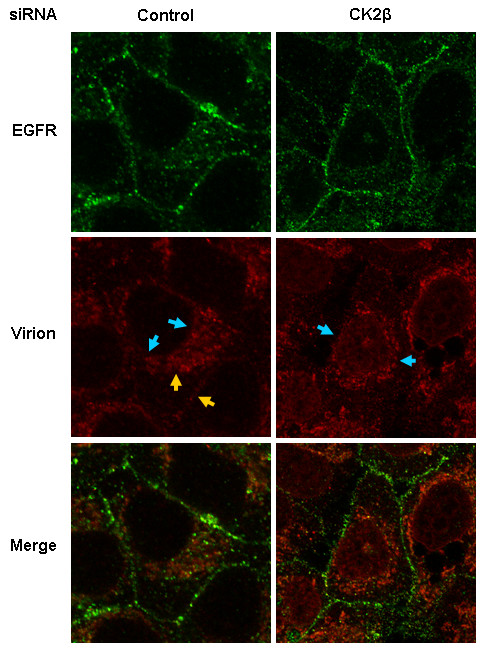
**siRNA-mediated CK2β silencing accelerates virus entry**. A549 cells were transfected with either control or CK2β siRNA for 48 h. They were then inoculated at an MOI of 50, chilled at 4°C for 30 min, and incubated at 37°C for 30 min. Influenza A virions (red) were detected with a goat anti-IVA (H1N1) polyclonal antibody, and an epidermal growth factor receptor (EGFR)-specific mAb (green) was used as a cell membrane marker. Images were taken with a confocal laser scanning microscope at 100× magnification. Blue arrows indicate virus accumulation at the nuclear membrane; yellow arrows indicate virus accumulation in the cytoplasm and at the cell membrane.

Having shown that 1) CK2β activation is inhibited during IVA replication and 2) CK2β silencing results in increased virus titers, we next studied the effect of CK2β gene silencing on the initial step of the viral replication cycle: virus entry. We transfected cells with either control or CK2β siRNA for 48 h. Cells were then inoculated with a high dose of virus (MOI = 50), incubated at 4°C for 30 min to block virus entry while binding to the cell surface, and incubated at 37°C for 30 min to allow virus entry. The cell membranes were then stained with an antibody specific for epidermal growth factor receptor (EGFR) and examined by confocal microscopy. Virus entry was slowed in control siRNA-transfected cells but not in CK2β siRNA-transfected cells, where they had accumulated at the nuclear membrane (predominantly) and in the nucleus 30 min after temperature shift (Figure 4). Although most viruses localized at the nuclear membrane of control siRNA-transfected cells, significantly less viral antigen was detected within the nucleus, and a large proportion of the virus population still remained in the cytoplasm and at the cell membrane (small fraction) (Figure 4). Therefore, internalization and nuclear import of virus are substantially greater when CK2β protein is absent or reduced.

### Inhibition of CK2β expression increases viral protein quantity

**Figure 5:**
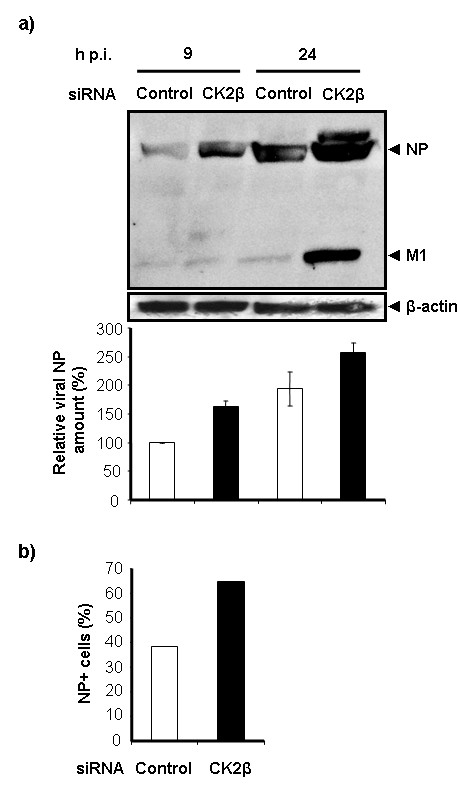
**siRNA-mediated silencing of CK2β leads to higher viral protein content**. (a) A549 cells were transfected with either control (white bars) or CK2β (black bars) siRNA. After 48 h, cells were inoculated at an MOI of 1 and incubated for 9 h or 24 h before Western blot analysis. Viral proteins were detected with a goat anti-IVA (H1N1) polyclonal antibody. Loading was controlled with a mAb against β-actin. Bands of viral protein were quantified as a percentage of control values normalized to the loading control. Shown are the mean ± SE from 3 independent experiments. (b) Cells were treated as above. The percentage of NP-expressing cells was measured at 24 h p.i. by flow cytometry (FACS) using an anti-NP mAb. The experiments were performed in duplicate.

We next examined whether CK2β gene silencing affects viral protein synthesis. Cells were transfected with either control or CK2β siRNA for 48 h, inoculated (MOI = 1) for 9 h or 24 h, and lysed for Western blot analysis. Total amount of viral protein was detected by using an antibody specific for IVA of the H1N1 subtype. As NP was the most heavily expressed of the viral proteins, we quantified the NP bands. The value obtained at 9 h p.i from cells transfected with control siRNA was assigned a value of 100%. NP production increased in both control and CK2β siRNA-transfected cells between 9 h and 24 h p.i. (Figure 5a).

However, a substantially larger amount of viral NP protein was synthesized in CK2β siRNA-transfected cells than in control-transfected cells at both time points (Figure 5a). While the expression of a late viral protein (matrix protein, M1) differed only slightly at 9 h p.i. between cells transfected with CK2β and control siRNA, M1 expression was higher in CK2β siRNA-transfected cells than in control-transfected cells at 24 h p.i. (Figure 5a).

To confirm that cells transfected with control siRNA and with CK2β siRNA differed in their viral protein content, we measured expression of NP protein in transfected cells at 24 h p.i. (MOI = 1) by FACS. Markedly more NP was produced in cells transfected with CK2β siRNA than in control siRNA-transfected cells (Figure 5b). 64% of the cell population transfected with CK2β siRNA expressed NP, whereas only 38% of the control siRNA-transfected cells were NP^+ ^(Figure 5b). This finding supports the results of our Western blot analysis and shows higher production of viral NP protein in CK2β siRNA-transfected cells.

### Inhibition of CK2β expression does not affect nuclear RNP export

**Figure 6:**
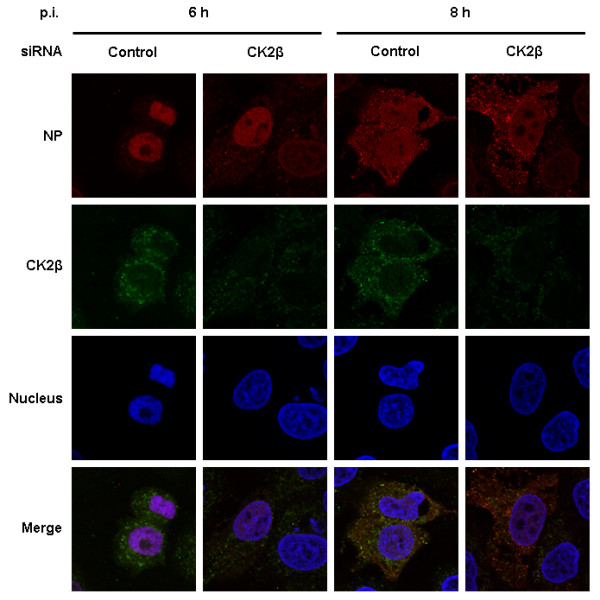
**siRNA-mediated CK2β silencing does not affect nuclear RNP export**. A549 cells were transfected with either control or CK2β siRNA for 48 h, inoculated with virus at an MOI of 1, and incubated for an additional 6 h or 8 h. The RNP complexes and CK2β protein were stained with a goat anti-NP antibody (red) and an anti-CK2β mouse monoclonal antibody (green), respectively. The nucleus was counterstained with TO-PRO-3 (blue). Intracellular RNP localization was analyzed at the indicated time points by confocal laser scanning microscopy (100× magnification).

Next, we investigated the contribution of CK2β to the intracellular localization of the viral RNP complexes. Cells were transfected with either control or CK2β siRNA for 48 h and then inoculated (MOI = 1) for 6 h and 8 h, the time points at which optimal nuclear export of RNP usually occurs [29,32,42]. Viral RNP complexes were stained with an anti-NP antibody, and CK2β protein was stained with anti-CK2β antibody. Confocal microscopy demonstrated a reduced amount of CK2β protein at 6 h and 8 h p.i in cells transfected with CK2β siRNA. In cells transfected with control siRNA, the majority of CK2β was found in the cytoplasm (Figure 6). At 6 h p.i. in cells transfected with CK2β siRNA, the viral RNP complexes were localized predominantly in the nucleus, with a small fraction in the cytoplasm. However, there was no substantial difference in nuclear RNP export in cells transfected with CK2β and control siRNA. Further, at 8 h p.i. viral RNP complexes were distributed in both the cytoplasm and the nuclei of control and CK2β siRNA-transfected cells (Figure 6), showing that the nuclear-cytoplasmic transport of viral RNP complexes is not affected by CK2β or by its absence.

### Inhibition of CK2β expression does not influence viral polymerase activity

**Figure 7:**
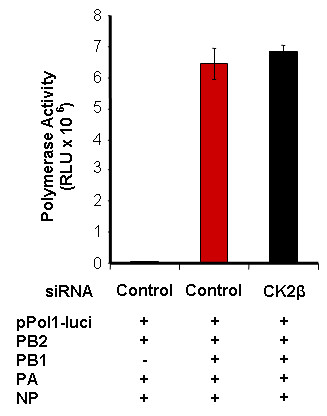
**siRNA-mediated CK2β gene silencing does not influence viral polymerase activity**. Polymerase activity was assayed in a minigenome system, using a viral UTR-driven luciferase reporter gene. A549 cells were transfected with either control or CK2β siRNA for 48 h, then co-transfected with plasmids containing the PB2, PB1, PA, and NP genes plus a luciferase reporter plasmid. Cells not transfected with the PB1 plasmid were used as negative controls. After 24 h, polymerase (luciferase) activity was assayed in cell extracts. Results represent the mean ± SE of 3 independent experiments.

We used a minigenome (RNP-like) system to evaluate whether CK2-mediated phosphorylation of PA and NP influences viral polymerase activity. Cells were transfected with either control or CK2β siRNA for 48 h and then co-transfected with the pol I-driven plasmid encoding the luciferase gene and pol I/pol II-responsive plasmids that express the viral PB2, PB1, PA, and NP proteins. After incubation for 24 h, luciferase activity (indicative of viral polymerase activity) was assayed in cell extracts. Transfection without the PB1 plasmid revealed negligible background luciferase expression. Inhibition of CK2β expression did not substantially influence viral polymerase activity, which was only slightly greater after CK2β siRNA treatment than after control siRNA treatment (Figure 7). The results indicate that the CK2β-mediated phosphorylation of PA and NP is not required for regulation of viral polymerase activity.

## Discussion

The molecular mechanisms of influenza virus multiplication and the interactions between viral and cellular gene products determine host susceptibility to infection and disease. We have demonstrated that the regulatory β-subunit of the cellular protein kinase CK2 inhibits IVA replication in A549 human epithelial lung cells. Importantly, pulmonary cells are among the first to be targeted by respiratory-tract pathogens, such as influenza viruses. When activated by infection, they can participate in lung inflammation by producing various soluble factors, including cytokines and by recruiting other cells [43].

siRNAs are widely used to study the function of cellular genes *in vitro *and *in vivo*. We found that this tool efficiently diminished CK2β expression in human lung cells without affecting the expression of CK2α protein. Cellular CK2β activation (phosphorylation) was upregulated during virus binding and internalization but diminished when virus replication began. Interestingly, inhibition of CK2β translation increased virus titers, suggesting that expression of this cellular kinase is inconsistent with influenza virus replication. It is noteworthy that virus titers in the absence of CK2β were inversely proportional to the MOI used for inoculation (i.e., the lower the MOI, the higher the titer); titers showed the greatest increase in cells inoculated at an MOI of 0.001. At this low MOI, the multiple replication cycles are required to infect the entire cell population leading to stronger differences in virus titers. Prevention of CK2β phosphorylation with a CK2 inhibitor also resulted in increased virus titers. In contrast, stimulation of CK2β with polyamine reduced virus titers, strengthening the evidence that CK2β activation negatively influences virus replication. This finding will facilitate further studies to understand the role of CK2β in viral biology. We hypothesize that the absence of CK2β reduces the activity of the CK2α subunit, thereby wholly or partially inactivating the heterotetrameric holoenzyme and providing a viral replication advantage. Our findings are compatible with a model in which influenza viruses have acquired the ability to exploit or impede host cell responses to support viral propagation. Several host factors other than CK2β have also been shown to possess anti-influenza virus activity. For example, inhibition of JNK signaling reportedly increases cellular virus yields [44].

Moreover, Mx1-induced expression of type I interferons efficiently protects mice against highly pathogenic H5N1 influenza virus by inhibiting viral polymerase activity, thereby reducing virus titers in the lung and liver [45]. Thus, to replicate optimally within the infected host, influenza viruses may employ "virus-supportive" cellular gene products while concomitantly suppressing the expression of genes that exert an antiviral effect.

Biochemical and genetic evidence indicates that CK2 participates in the maintenance of cell viability and plays a global anti-apoptotic role [2,46]. Treatment of Jurkat cells with a specific CK2 inhibitor induces apoptosis [47]. The anti-apoptotic effect of CK2 is at least partially mediated by upregulation of the Akt/PKB pathway [48]. Early studies demonstrated that overexpression of the anti-apoptotic protein Bcl-2 impairs influenza virus replication and is associated with a mis-glycosylation of the viral surface protein hemagglutinin [49]. Another study showed that when activation of caspase 3 (a major apoptosis effector) is blocked, export of nuclear RNP is retarded and virus titers are reduced [50]. These observations indicate that induction of apoptosis plays a predominantly antiviral role but can also support influenza virus replication. Thus, the expression of CK2 protein kinase may prevent virus propagation by controlling activation of apoptotic pathways and thereby reducing virus titers. On the other hand, previous studies showed that CK2α activity is essential for influenza virus budding in MDCK cells [51], although the direct effect on virus titers was not reported. Hence, it remains to be determined why the α and β subunits of CK2 appear to have contradictory effects on the replication of influenza virus, although these discrepancies may reflect the different cell lines used in the studies.

Export of viral RNPs from the nucleus is a key step in the influenza virus infectious cycle. Inhibition of CK2β expression did not affect the nuclear export of viral RNP complexes at 6 h and 8 h p.i.; thus, nuclear RNP export does not appear to be responsible for the increase in virus titers. Instead, influenza virus interacts with and employs other host factors, such as ERK [29,32], CRM1 [52], and caspase 3 [50] to accomplish the process of nuclear RNP export. This is supported by our data showing that virus-induced ERK activation is not affected in either presence or absence of CK2β (data not shown). In contrast, Bcl-2 appears to be involved in negatively regulating this mechanism: in cells expressing Bcl-2 the viral RNP complexes accumulate around the nuclear membrane, whereas they are distributed throughout the cytoplasm in control cells [53]. We also determined the effect of CK2β silencing on viral polymerase activity in an RNP-like construct. Although PA and NP are the 2 known phosphoproteins [33,36,54] in the RNP complexes and are predicted to possess CK2 phosphorylation sites (NetPhosK 1.0) [55], presence or absence of the CK2β protein did not affect viral polymerase activity in this system. Hence, CK2-mediated phosphorylation does not appear to be essential for viral polymerase activity.

Many viruses use receptor-mediated endocytosis to enter a target cell [56,57]. Activation of cellular gene products such as PKCβII and phosphatidylinositol-3-kinase are suggested to be involved in this process [30,58]. PKCβII plays a role in both influenza virus entry and EGFR trafficking and degradation. In cells lacking functional PKCβII, viral RNP complexes and EGFR were trapped in late endosomal compartments [30]. In contrast, in cells lacking CK2β protein, a very early step in virus uptake from the cell membrane into the nucleus was substantially enhanced. Our immunofluorescence analysis revealed enhanced virus internalization and accelerated virus transport from the cytoplasm to the nucleus in cells with reduced CK2β expression. *CK2*β gene silencing appeared to promote influenza virus entry by reducing the inhibitory effect of CK2β. However, further analysis is required to fully decipher how and at what level CK2β inhibits this early stage of viral replication.

Reduced or absent CK2β expression substantially enhanced the cellular viral protein content, suggesting that CK2β may interfere with viral protein synthesis at the molecular level, e.g., replication (viral RNA ↔ cRNA), transcription (cRNA → mRNA), or translation. On the other hand, a considerably greater quantity of viral NP was detected at 24 h p.i. than at 9 h p.i., indicating the progressive spread of infection during that time. We hypothesize that while the viral protein synthesis rate per cell remains constant, loss of the CK2β protein seems to increase the susceptibility of A549 cells to influenza virus infection in the whole cell population. However, further investigation is needed to determine how CK2β absence increases cellular viral protein and why this increase does not significantly affect viral polymerase activity. The effect of *CK2*β gene silencing on accumulation of viral RNA, cRNA, and mRNA in infected cells should be examined in more detail. Overall, siRNA-mediated *CK2*β gene silencing appears to increase cell susceptibility to influenza virus infection resulting in accelerated virus entry process and larger viral protein quantity, and these effects in turn contribute to higher virus titers. However, the molecular mechanisms remain to be elucidated.

## Conclusion

Our results are the first to demonstrate a functional link between the CK2β protein and regulation of IVA replication. In contrast to virus-induced upregulation of many cellular kinases, IVA appears to constrain CK2β activation during viral replication. We found that *CK2*β gene silencing significantly increases virus titers in A549 cells, explaining the advantage of viral inhibition of CK2β. Treatment of cells with a specific CK2 inhibitor also increased virus titers, whereas stimulation of CK2β reduced virus titers, suggesting that CK2β activation negatively affects virus replication. *CK2*β gene silencing impaired neither nuclear export of viral RNP complexes nor viral polymerase activity. However, it appears to increase cell susceptibility to influenza virus infection and thereby accelerates virus entry process and boosts the total viral protein content. siRNA-mediated *CK2*β gene silencing may be useful in improving the yield of vaccine-strain influenza virus. Clinically, it may be possible in the future to introduce a construct expressing a constitutively active CK2β into influenza-infected hosts as a therapeutic intervention.

## Materials and methods

### Cells, virus, and infection

The human epithelial lung cell line A549 was purchased from the American Type Culture Collection (Rockville, MD) and was cultured in FK12 medium containing 10% (v/v) fetal bovine serum (FBS) and 1% (v/v) antibiotics (penicillin/streptomycin). Madin-Darby canine kidney (MDCK) cells were maintained in Dulbecco's Modified Eagle's Medium (DMEM) supplemented with 10% (v/v) fetal calf serum (FCS) and 1% (v/v) antibiotics (penicillin/streptomycin). All cells were cultivated at 37°C in a humidified atmosphere containing 5% CO_2_. The H1N1-subtype human influenza virus A/Puerto Rico/8/34 (PR/8) was generated by 8-plasmid reverse genetics [59] and used at the indicated multiplicity of infection (MOI; determined in MDCK cells) in the presence of TPCK trypsin (1:6000).

### Virus titration (plaque assay)

Confluent monolayers of MDCK cells in 35-mm dishes were inoculated with 10-fold dilutions of PR/8 influenza virus in DMEM with 3% bovine serum albumin (BSA) and antibiotics and incubated at 37°C for 1 h. The inoculum was removed, and cells were washed with PBS and overlaid with 2-fold DMEM containing 1.8% bacto-agar, 0.2% BSA, and 2 μg/ml TPCK trypsin. After 3 days of incubation at 37°C, cells were stained with 0.1% crystal violet in 10% formaldehyde solution and the plaques were counted.

### Cell treatment with CK2 inhibitor and activator

To determine the effect of CK2 inhibition, confluent monolayers of A549 cells in 35-mm dishes were treated with 5 μM 2-dimethylamino-4,5,6,7-tetrabromo-1H-benzimidazole (DMAT; Calbiochem, San Diego, CA) or with solvent (DMSO) (Sigma-Aldrich, St. Louis, MO) 1 h prior to infection. To determine the effect of CK2β activation, cells were treated with 300 μM spermine (Sigma-Aldrich) or with H_2_O 1 h prior to infection. Cells were then inoculated at MOI = 5 in the presence of DMAT or DMSO. Virus-containing supernatants were harvested 24 h post-infection (p.i.) and virus titers were determined by plaque assay.

### Transient siRNA transfection

Human CK2β siRNA (Santa Cruz Biotechnology, Santa Cruz, CA) is a pool of 3 target-specific 20- to 25-nt siRNAs designed to silence CK2β gene expression in human cells. A549 cells (grown in 35-mm dishes) were transfected with siRNA duplex (1 μg) by using Lipofectamine 2000 (Invitrogen, New Brunswick, NJ). The control siRNA (Santa Cruz Biotechnology) is a nontargeting 20- to 25-nt siRNA that does not cause specific degradation of any known cellular mRNA. Cells were transfected for 48 h, washed with FK12 medium without supplements, then inoculate at the indicated MOIs and incubated for different time periods, depending on the purpose of the experiment. Transfection efficiency monitored by fluorescein-conjugated control siRNA (Santa Cruz Biotechnology) varied up to 85% (flow cytometry analysis) according to the maximum protein level reduction.

### Cell proliferation assay

A549 cells were grown in 96-well dishes and transfected with 1 μg of CK2β siRNA or control siRNA for 24 h, 48 h, or 72 h at 37°C, 5% CO_2_. The cell medium was then replaced with fresh medium. After further incubation for 1 h, the medium was replaced with 200 μl of FK12 medium containing 10% (v/v) FBS and tetrazolium bromide (MTT; 175 μg mL^-1^, Sigma-Aldrich). After 90 min, the incubation medium was replaced with PBS/4% paraformaldehyde (PFA), and cells were fixed for 30 min at room temperature (RT). Cells were air dried and, after addition of isopropanol (150 μL/well), plates were vigorously agitated for 10 min. The plates were photometrically analyzed at 550 nm in an enzyme-linked immunosorbent assay reader (Bio-Rad Microplate Reader, Model 680). Sixteen samples from each group were measured, and the mean and standard error was calculated. Cell morphology was analyzed by confocal laser scanning microscopy after transfection with either siRNA.

### Western blot analysis

Cell lysate was cleared by centrifugation, and total protein concentration was determined by the Bradford assay before separation by SDS-PAGE. A rabbit polyclonal antibody that detects phosphorylated CK2β (P-S209) (1:200; abcam, Cambridge, MA) was used to determine CK2β activity. Expression of CK2α and CK2β were detected with anti-CK2α (1:200; Calbiochem) and anti-CK2β (1:500; Calbiochem) mouse monoclonal antibody, respectively. Anti-β-actin mouse monoclonal antibody (1:500; Santa Cruz Biotechnology) was used to detect β-actin expression as a protein-loading control. Proteins recognized by mAbs were further analyzed with horseradish peroxidase-conjugated, species-specific secondary antibodies (1:2000; Jackson ImmunoResearch, West Grove, PA) and a standard enhanced chemiluminescence reaction (Amersham Biosciences, Piscataway, NJ). Specific bands were quantified by using the PC-BAS software package (Fuji, Kanagawa, Japan). To detect synthesis of viral proteins, cells were incubated with a goat anti-IVA (H1N1) polyclonal antibody (1:500; Biodesign International, Saco, ME).

To detect ERK activation, cells were transfected with either control or CK2β siRNA for 48 h and were then infected at an MOI of 5 and further incubated for 4, 6, or 8 h. After Western blot analysis, ERK activation was measured with a mAb specific for phosphorylated ERK (1:500; Santa Cruz Biotechnology). ERK2 (1:500; Santa Cruz Biotechnology) served as a protein loading control.

### FACS analysis

After 48 h of transfection with either siRNA, cells were infected at MOI = 1 and incubated for 24 h. Cells were detached with trypsin, fixed in PBS/4% PFA, permeabilized with 1% Triton X-100, and incubated with FITC-conjugated mouse anti-NP mAb (clone IA52, 1:500; Argene Inc) in PBS/3% BSA for 30 min on ice. The percentage of NP-expressing cells was determined by FACS analysis using (FACSCalibur, BD Biosciences, Rockville, MD).

### Indirect immunofluorescence assay and confocal laser scanning microscopy

A549 cells grown on collagen-coated glass coverslips (BD, Franklin Lakes, NJ) were transfected with either control or CK2β siRNA for 48 h, then infected at MOI = 1 and incubated further for 6 h and 8 h. Cells were washed with PBS at the indicated time points p.i. and fixed with 4% PFA in PBS for 30 min at room temperature or overnight at 4°C. Cells were permeabilized with 1% Triton X-100 in PBS for 10 min and incubated with a goat anti-NP antibody, clone G150 (1:100; St Jude Children's Research Hospital) and anti-CK2β mouse monoclonal antibody (1:100; Calbiochem) in PBS/3% BSA for 1 h for analysis of the nuclear export of RNP complexes and the amount of expressed CK2β protein. The AlexaFluor488-conjugated goat anti-mouse antibody and AlexaFluor594-conjugated donkey anti-goat antibody (1:200; Molecular Probes, Invitrogen, Carlsbad, CA) were used as secondary antibodies. Cells were washed with PBS and then with double-distilled water and mounted with para-phenylene diamine (Sigma-Aldrich) containing 500 nM TO-PRO-3 (Molecular Probes, Invitrogen) for nuclear staining. Fluorescence was visualized with a confocal laser scanning microscope (LSM 510 META, Carl Zeiss, Germany).

To study virus entry, cells were infected at a MOI = 50, chilled at 4°C for 30 min, and incubated at 37°C for 30 min. Cells were permeabilized, fixed, and incubated with a combination of goat anti-IVA (H1N1) polyclonal antibody (1:100) (Biodesign International) and mouse anti-epidermal growth factor receptor (EGFR) mAb (1:100; Santa Cruz Biotechnology). Alexa594-conjugated donkey anti-goat and AlexaFluor488-conjugatedgoat anti-mouse antibodies (Molecular Probes) were used as secondary antibodies.

### Luciferase assays

Subconfluent monolayers of A549 cells (7.5 × 10^5 ^cells in 35-mm dishes) were transfected with CK2β siRNA or control siRNA for 48 h. Cells were then transfected with 2 μg of luciferase reporter plasmid (enhanced green fluorescent protein (EGFP) open reading frame in pHW72-EGFP substituted with the luciferase gene[60]) and a mixture of the PB2 (1 μg), PB1 (1 μg), PA (1 μg), and NP (2 μg) plasmids of A/Puerto-Rico/8/34 (H1N1) virus. After 24 h, cell extracts were prepared in 500 μL of lysis buffer, and luciferase levels were assayed with a luciferase assay reagent kit (Promega, Madison, WI) and BD Monolight 3010 luminometer (BD Biosciences).

## Competing interests

The authors declare that they have no competing interests.

## Authors' contributions

HM performed most of the experiments and wrote the manuscript. H–LY made the luciferase reporter plasmid. CS and RGW contributed to scientific ideas and analysis of the data. All authors reviewed the manuscript, and all author approved the final version.
